# Prof. Allen J. Smith, M. D.

**Published:** 1892-10

**Authors:** 


PROF. ALLEN J. SMITH, M. D., Bacteriologist and Microscopist, Medical Department University of Texas, is hereby announced as Associate Editor of the JOURNAL, and will contribute to the editorial department, as well as to the department of clinical reports, etc. We are also pleased to announce that Professors J. E. Thompson, M. D., Professor of Surgery, and Wm. Keiller, M. D., Professor of Anatomy (also of the Texas Medical College, Galveston), have promised contributions to the JOURNAL occasionally during the fall and winter.

